# Two-Year Outcomes of Visian Implantable Collamer Lens with a Central Hole for Correcting High Myopia

**DOI:** 10.1155/2018/8678352

**Published:** 2018-07-03

**Authors:** Zhipeng Yan, Huamao Miao, Feng Zhao, Xiaoying Wang, Xun Chen, Meiyan Li, Xingtao Zhou

**Affiliations:** ^1^Department of Ophthalmology, Third Hospital of Hebei Medical University, Shijiazhuang, Hebei, China; ^2^Department of Ophthalmology and Optometry, Eye and ENT Hospital, Fudan University, Shanghai, China; ^3^NHC Key Laboratory of Myopia, Fudan University, Shanghai, China; ^4^Shanghai Research Center of Ophthalmology and Optometry, Shanghai, China

## Abstract

**Purpose:**

To investigate two-year outcomes of Visian Implantable Collamer Lens with a central hole (ICL V4c) implantation for correcting high myopia.

**Methods:**

Sixty-one eyes of 32 patients went through ICL V4c implantation. Safety, efficacy, predictability, and intraocular pressure were evaluated 2 years postoperatively. Anterior chamber volume (ACV), anterior chamber depth (ACD), anterior chamber angle width (ACAW), and vault were measured using a Scheimpflug tomography imaging system.

**Results:**

At 2 years, the spherical equivalent refraction decreased from preoperative −14.62 ± 4.29 D to −0.90 ± 0.95 D, with 79% of the eyes within ±0.50 D and 98% within ±1.00 D of the intended correction. The efficacy index was 1.03 ± 0.23, and the safety index was 1.24 ± 0.26. Corrected distance visual acuity (CDVA) remained unchanged in 23% of the eyes, 75% gained one or more lines of CDVA, and 2% lost one line. The ACV, ACD, and ACAW, respectively, decreased from 193.28 ± 29.15 mm^3^, 3.15 ± 0.23 mm, and 36.51 ± 6.54 degree to 112.48 ± 17.01 mm^3^, 2.99 ± 0.23 mm, and 22.54 ± 5.27 degree (*p*=0.0008, 0.008, and  0.0003, resp.). Intraocular pressure was 15.39 ± 2.88 mmHg before surgery and was 15.86 ± 4.11 mmHg at 2 years (*p*=0.11).

**Conclusion:**

Implantation of ICL V4c is a safe, effective, and predictable procedure for correcting high myopia. Reduction of anterior chamber space after surgery did not induce intraocular pressure increase during the 2-year follow-up.

## 1. Introduction

The high prevalence of high myopia in Chinese young adults has become a social public health issue in China [1]. Currently, laser refractive surgery remains the most common surgical procedure to correct myopia. However, it has limitations in correcting high myopia. The posterior chamber phakic intraocular lens implantation could correct a wider range of myopia, and it has become an important surgical option for patients who are unsuitable for corneal refractive surgeries.

The Visian Implantable Collamer Lens V4c (ICL V4c) is a new generation of posterior chamber phakic intraocular lens, which is capable to correct up to −18.00 D refraction [2]. Compared with the traditional ICL V4, the ICL V4c was designed with a 0.36 mm central hole, which improves aqueous humor circulation and obviates the need for peripheral iridotomy or iridectomy. To fully understand the safety of this technique, the present 2-year study evaluated refractive outcomes, anterior segment changes, and lens position after ICL V4c implantation in patients with myopia of −8.00 D or higher.

## 2. Patients and Methods

This is a nonrandomized prospective study. All the patients underwent routine ophthalmic examinations at the Refractive Surgery Center of the Department of Ophthalmology, Eye and ENT Hospital of Fudan University (Shanghai, People's Republic of China), and met the surgical requirements for ICL V4c (STAAR Surgical Company, Monrovia, California, USA) implantation. The inclusion criterion was with preoperative spherical equivalent (SE) of −8.00 D or higher. Exclusion criteria were corneal endothelial cell density (ECD, noncontact specular microscopy, SP-2000P, Topcon Corporation, Japan) less than 2000 cell/mm^2^, a history of ocular surgery, cataract, glaucoma, amblyopia, retinal detachment, neuroophthalmic diseases, and ocular inflammatory diseases. Thirty-two patients (61 eyes) with 14 males and 18 females were consecutively enrolled, and none was lost at two years. Patient demographic data and ICL V4c characteristics are listed in Table 1. Their mean age was 30.87 ± 8.03 years (range, 20 to 45 years). The mean preoperative SE was −14.62 ± 4.29 D (range, −8.00 D to −25.75 D), and 23% (14 eyes) of the eyes had preoperative SE over −18.00 D.

The study adhered to the tenets of the Declaration of Helsinki and was approved by the Ethics Committee of the Eye and ENT Hospital, Fudan University. All patients signed their informed consent after a detailed explanation of the possible risks and benefits of the study.

## 3. Surgical Technique

The ICL V4c is a plate-haptic single-piece intraocular lens made of Collamer. It has a central convex-concave optical zone and incorporates a forward vault to minimize contact with the crystalline lens. A 360 *μ*m central hole was included to improve aqueous humor circulation, which eliminates the need for preoperative laser peripheral iridotomy. The ICL V4c corrects −0.50 D to −18.00 D myopic spherical refraction and up to −5.00 D cylindrical refraction. There are 4 sizes: 12.1 mm, 12.6 mm, 13.2 mm, and 13.7 mm. Power calculation of the ICL V4c was performed by the manufacturer (STAAR Surgical) using a modified vertex formula, according to the provided preoperative refractive parameters. The size of the implanted ICL V4c was determined based on the white-to-white horizontal corneal diameter and anterior chamber depth.

In the present study, the ICL V4c type (toric or nontoric) and the targeted spherical and cylindrical correction powers were designed as follows. Thirty-one eyes were implanted with toric ICL V4c. Of the 31 eyes, 2 eyes had SE over −18.00 D, in which one eye had cylindrical diopters that have been undercorrected and the other eye had both the cylindrical and the spherical diopters that have been undercorrected; three eyes had preoperative cylindrical diopters from −4.25 D to −5.50 D, and a cylindrical undercorrection of −1.25 D to −1.50 D was designed. Thirty eyes were implanted with nontoric ICL V4c. Of the 30 eyes, 12 eyes had preoperative SE over −18.00 D, in which 3 eyes had cylindrical diopters that have been undercorrected, 1 eye had spherical diopters that have been undercorrected, and 8 eyes had both the cylindrical and the spherical diopters that have been undercorrected; fifteen eyes tried spectacle lenses without cylindrical diopters being corrected before surgery, and the patients were satisfied with the corresponding corrected distance visual acuity, thus chose the nontoric ICL V4c.

ICL V4c implantation procedures were performed by an experienced surgeon (XZ). Pupils were dilated before surgery. After injection of 1% sodium hyaluronate into the anterior chamber, ICL V4c was then implanted via a 3.0 mm temporal corneal incision using an injector cartridge and then was placed in the posterior chamber. After that, the viscoelastic surgical agent was washed away using the balanced salt solution, and a miotic agent was instilled. Postoperative medications included antibiotic eye drops, nonsteroidal anti-inflammatory eye drops, steroidal eye drops, and artificial eye drops.

## 4. Measurements

The following parameters were recorded for analysis: uncorrected distance visual acuity (UDVA), corrected distance visual acuity (CDVA), and manifest refractions. The safety index was calculated as the ratio between the CDVA at 2 years and the corresponding CDVA before surgery, and the efficacy index was the ratio between the UDVA at 2 years and the corresponding CDVA before surgery. Intraocular pressure (IOP) was measured with a noncontact tonometer (NCT; Canon, Japan). The central anterior chamber depth (ACD), anterior chamber volume (ACV), anterior chamber angle width (ACAW), and the ICL V4c vault were measured using the Pentacam (Oculus, Germany) through a rotating Scheimpflug camera. The haptic positions of the ICL V4c lens implanted were measured using an ultrasound biomicroscopy (UBM, MD-300L, MEDA Co., Ltd., Tianjin, China). Patient satisfaction was assessed at 2 years. They were asked to rate if they were very satisfied, satisfied, not so satisfied, or very unsatisfied with their visual performance. And overall satisfaction score (ranging from 0 = very unsatisfied to 10 = the most satisfied) was also collected.

## 5. Statistical Analysis

Statistical analysis was performed using SAS V9.3 (Cary, NC, USA). For the sake of within-subject correlation, a linear mixed model was used to detect the differences of variables including ACD, ACV, ACAW, IOP, and vault between time points. Correlation analysis was used to evaluate the relationship between two continuous variables. A *P* value of less than 0.05 was considered statistically significant.

## 6. Results

All surgeries were uneventful, and no vision threatening complications were observed during the follow-up period. The mean IOP was 15.39 ± 2.88 mmHg before surgery and 15.86 ± 4.11 mmHg at 2 years (*p*=0.11). The mean ECD was 3251 ± 506 cell/mm^2^ before surgery and 3246 ± 522 cell/mm^2^ at 2 years (*p*=0.95).

### 6.1. Refractive Outcomes

**Figure 1:**
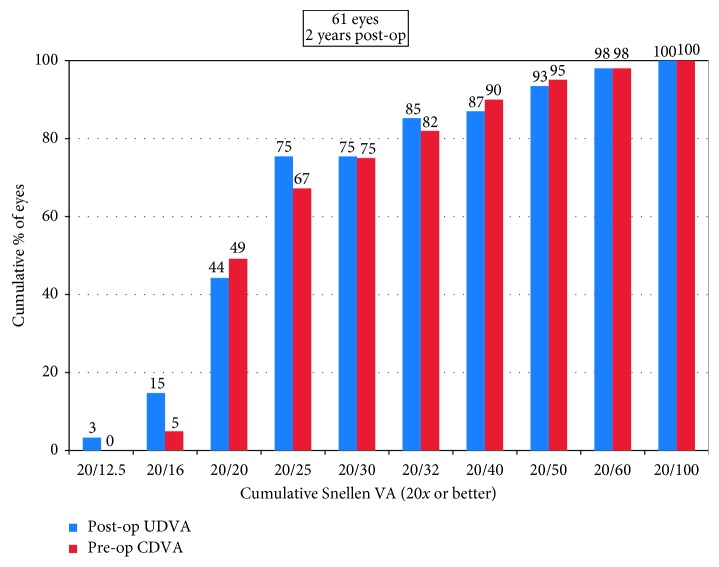
Cumulative percentage of the eyes attaining specified cumulative levels of uncorrected distance visual acuity (UDVA) 2 years after surgery.

**Figure 2:**
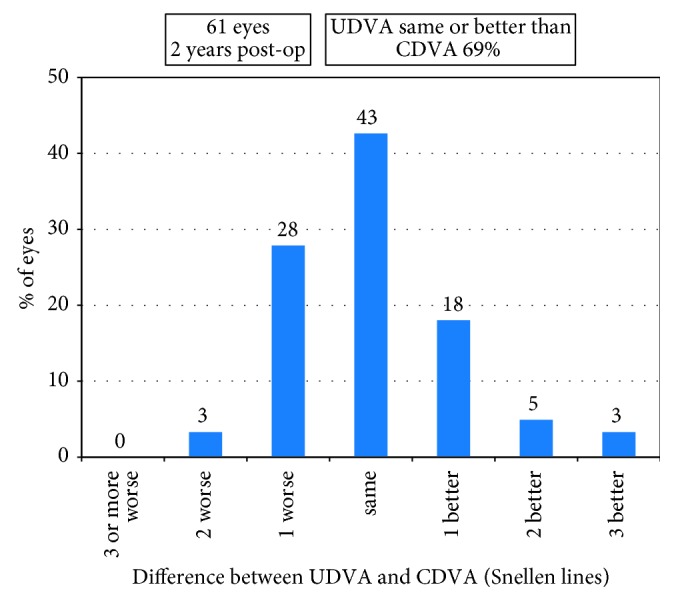
Percentage of the eyes comparing 2 years' postoperative uncorrected distance visual acuity (UDVA) and preoperative corrective distance visual acuity (CDVA).

**Figure 3:**
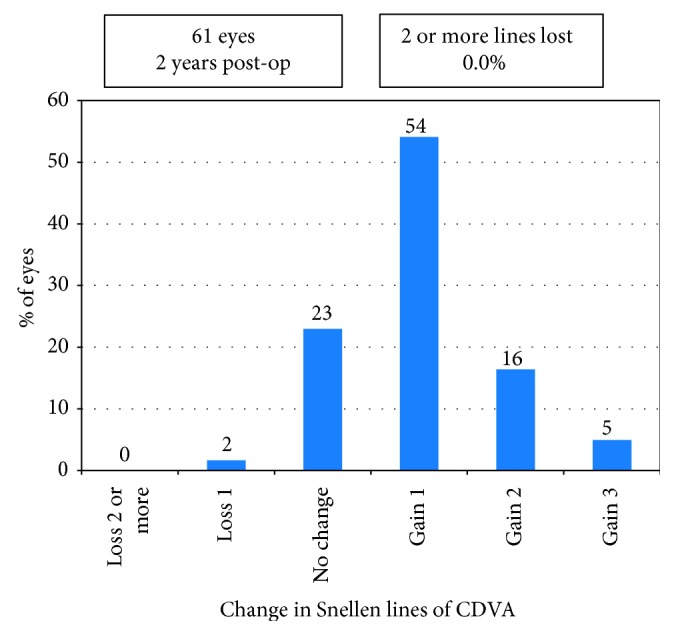
Gain and loss of corrected distance visual acuity (CDVA) 2 years postoperatively.

The decimal UDVA and CDVA were 0.84 ± 0.28 (range, 0.2–1.5) and 1.00 ± 0.27 (range, 0.2–1.5) at 2 years, respectively. Forty-six of the 61 eyes (75%) had postoperative UDVA of 20/25 or better (Figure 1). Forty-two eyes (69%) had postoperative UDVA same or better than preoperative CDVA (Figure 2). As shown in Figure 3, 14 eyes (23%) had postoperative CDVA which remained unchanged, 33 eyes (54%) gained one line, 13 eyes (21%) gained two or more lines of CDVA, and one eye (2%) lost one line of CDVA. The efficacy index (the ratio between postoperative UDVA and preoperative CDVA) was 1.03 ± 0.23 (range, 0.5 to 1.67). The efficacy index was 1.05 ± 0.20 or 1.02 ± 0.26 in the eyes with postoperative SE at 2 years within ± 0.50 D or over −0.50 D (*p*=0.65). The safety index (the ratio between postoperative CDVA and preoperative CDVA) was 1.24 ± 0.26 (range, 0.83 to 2.0).

**Figure 4:**
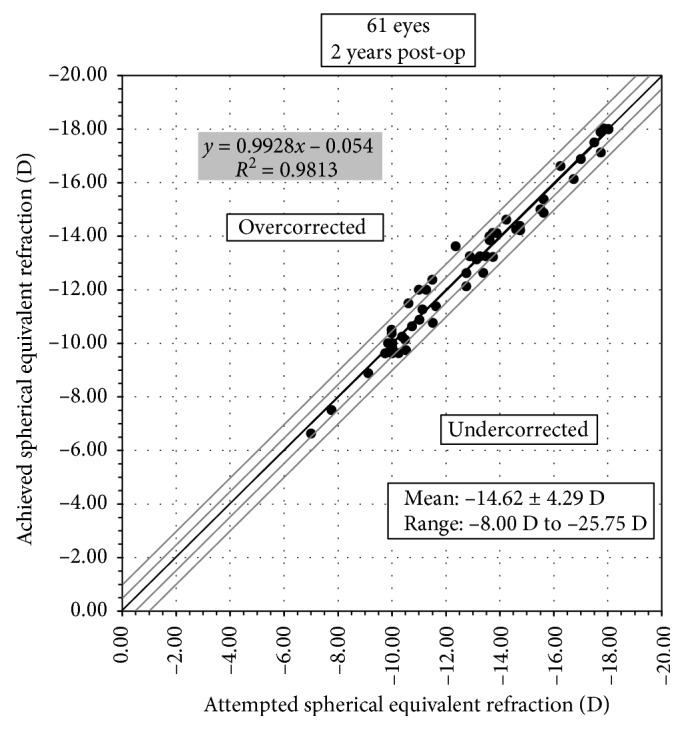
Attempted spherical equivalent refraction change versus the achieved spherical equivalent refraction change at 2 years.

**Figure 5:**
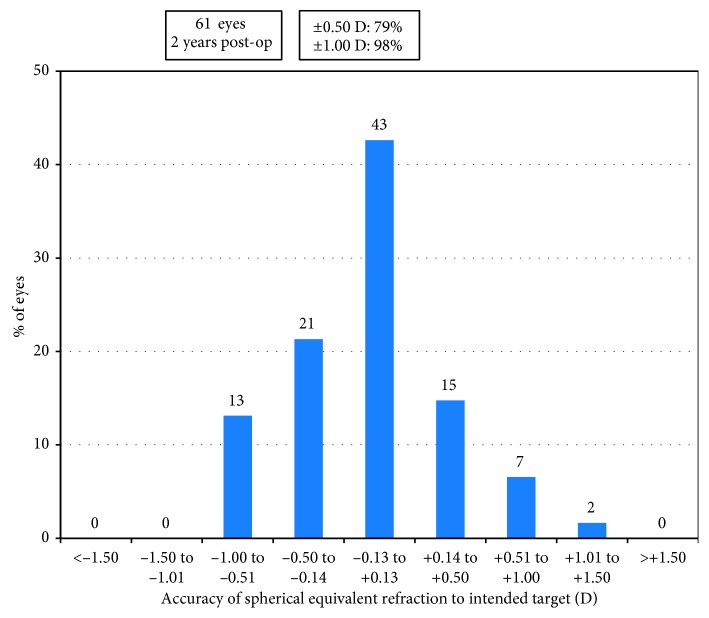
Percentage of the eyes attaining specified differences in attempted versus achieved correction at 2 years.

**Figure 6:**
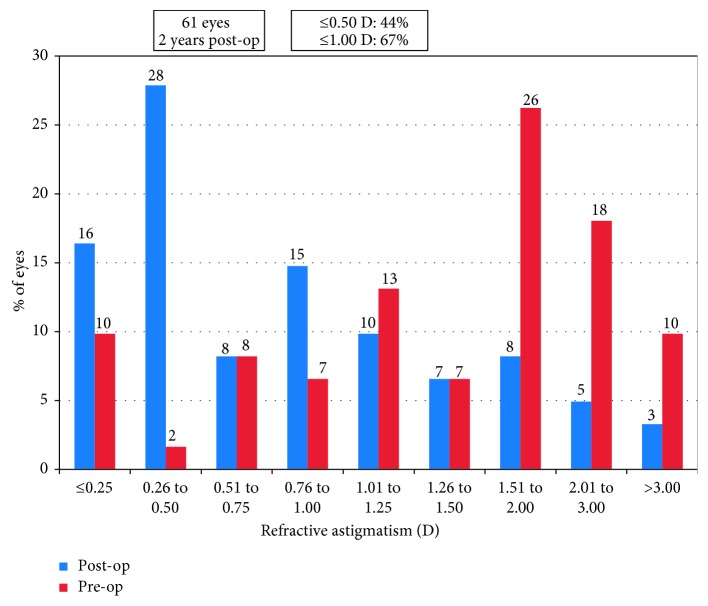
Percentage of the eyes within different astigmatism ranges before operation and at 2 years.

**Figure 7:**
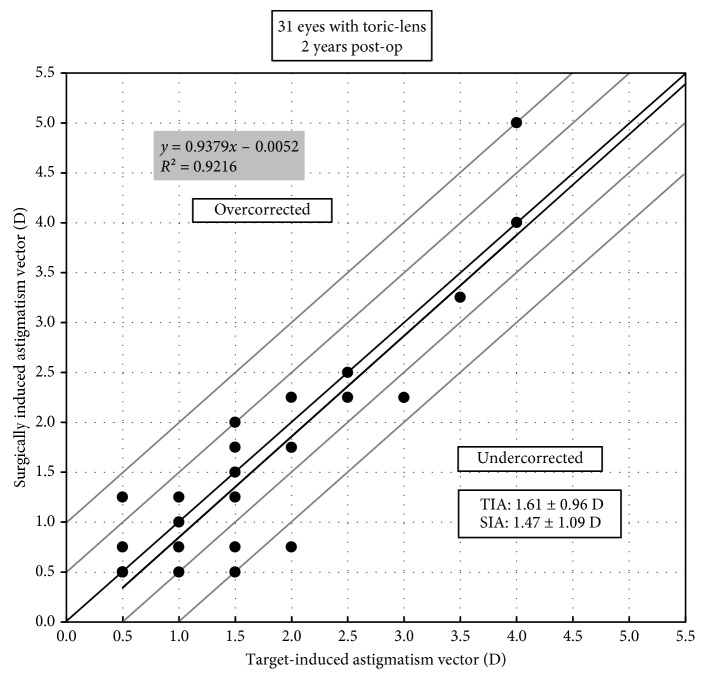
Target-induced astigmatism (TIA) versus surgically induced astigmatism (SIA) at 2 years in the eyes implanted with toric ICL V4c.

Before operation, 59 eyes (97%) had SE over −9.00 D, and 14 eyes (23%) had SE over −18.00 D. Mean SE decreased from preoperative −14.62 ± 4.29 D to −0.90 ± 0.95 D at 2 years (*p* < 0.0001). Mean astigmatism decreased from −1.82 ± 1.22 D to −0.98 ± 0.93 D (*p* < 0.001). A scatter plot with a best-fit line (*y* = 0.99*x* − 0.05, *R*^2^ = 0.98) of the attempted versus the achieved spherical equivalent correction is shown in Figure 4. At 2 years, 79% (48 eyes) of the eyes were within ±0.50 D and 98% (60 eyes) were within ±1.00 D of the intended correction (Figure 5). Before operation, the percentage of the eyes with astigmatism ≤0.50 D, ≤ 1.00 D, ≤2.00 D, and ≤3.00 D were 11.5%, 26%, 72%, and 90%, respectively, and at 2 years, they increased to 44%, 67%, 92%, and 97%, respectively (Figure 6). For the 31 eyes implanted with toric ICL V4c, a scatter plot with a best-fit line (*y* = 0.94*x* − 0.01, *R*^2^ = 0.92) of the target versus the surgically induced astigmatism is shown in Figure 7.

All patients were satisfied with their visual performance, and 71% were more than satisfied (very satisfied). The mean score of overall satisfaction was 9.27 ± 0.87 (range, 8 to 10). And all the patients chose “yes,” when asked “Would you consider recommending this operation to patients like you?”

### 6.2. Anterior Chamber Changes

**Figure 8:**
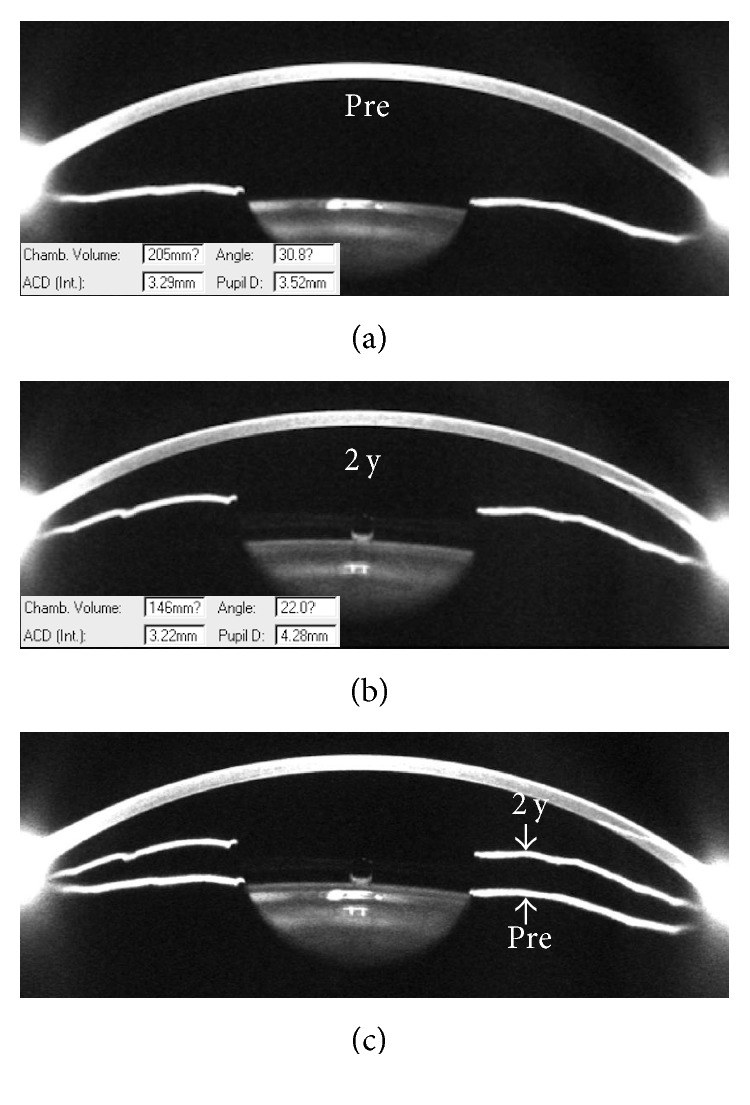
Pentacam sample images of the anterior chamber segment changes before and 2 years after the ICL V4c was implanted. Patient information: male, 31 years, left eye, with preoperative spherical equivalent refraction of −10.88 D (−10.50DS/−0.75DC × 130°), and a nontoric ICL V4c was implanted. Before surgery, the anterior chamber volume was 205 mm^3^, the central anterior chamber depth was 3.29 mm, and the anterior chamber angle was 30.8 degree (a). At 2 years, the anterior chamber volume was 146 mm^3^, the central anterior chamber depth was 3.22 mm, the anterior chamber angle was 22.0 degree, and the vault was 470 *μ*m (b). The merged image (c) of A and B showed that the iris was pushed forward after ICL V4c implantation.

Mean ACV was 193.28 ± 29.15 mm^3^, mean ACD was 3.15 ± 0.23 mm, and mean ACAW was 36.51 ± 6.54 degree before operation; they, respectively, decreased to 112.48 ± 17.01 mm^3^, 2.99 ± 0.23 mm, and 22.54 ± 5.27 degree at 2 years (*p*=0.0008, 0.008, and  0.0003, resp.).  Figure 8 showed an example of anterior chamber changes measured by Pentacam before operation and at 2 years postoperatively.

The postoperative ACD was significantly correlated with preoperative ACD (Spearman's rho, *r*=0.85, *p* < 0.0001). The postoperative ACV was significantly correlated with preoperative ACV (*r*=0.60, *p* < 0.0001). No significant relationship was found between postoperative and preoperative ACAW (*r*=0.21, *p*=0.10). IOP at 2 years was not significantly correlated with postoperative ACD (*r*=−0.04, *p*=0.76), ACV (*r*=0.09, *p*=0.50), or ACAW (*r*=0.18, *p*=0.17).

Mean vault at 1 day, 1 week, and 2 years was 426 ± 168 *μ*m, 463 ± 186 *μ*m, and 449 ± 167 *μ*m, respectively (*p*=0.72). Correlation analyses revealed that vault at 2 years was significantly correlated with postoperative ACAW (*r*=−0.67, *p* < 0.0001) and ACV (*r*=−0.36, *p*=0.004) but was of no significant relationship with ACD (*r*=0.20, *p*=0.13).

### 6.3. ICL V4c Haptics Positions

**Figure 9:**
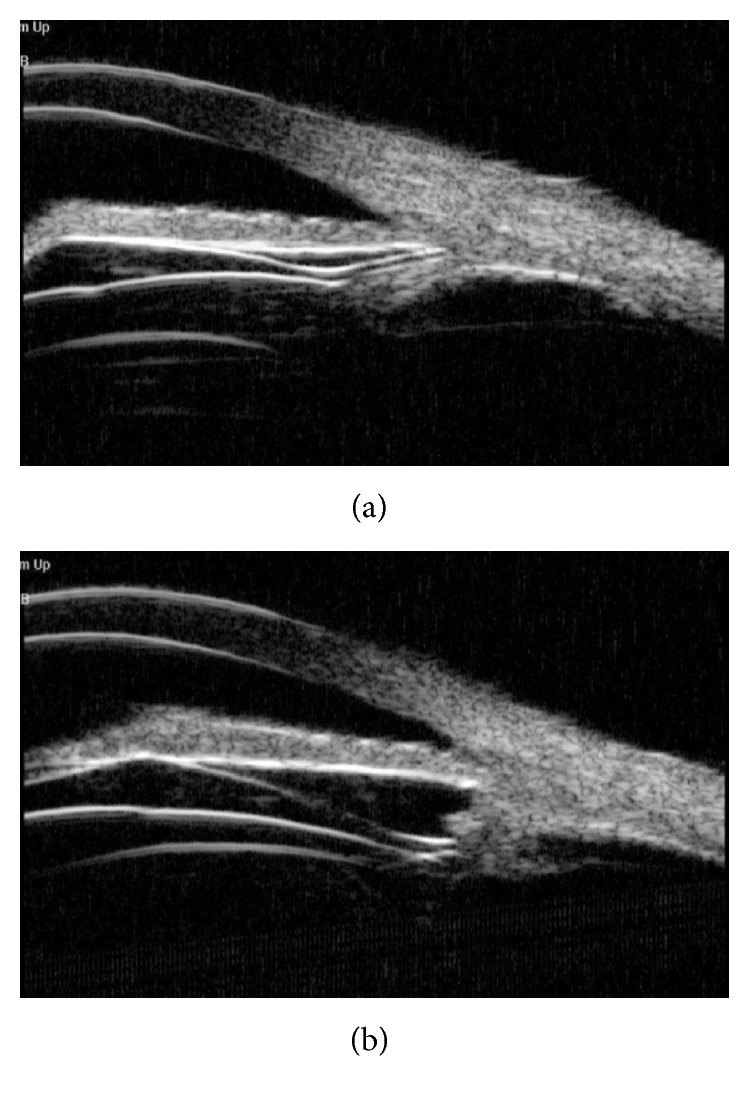
Ultrasound biomicroscopy sample images of the ICL V4c haptic position and its relationship with the ciliary body. The haptics were either located in the ciliary sulcus (a) or in the ciliary processes (b).

The positions of the ICL V4c haptics were examined in 46 eyes (24 patients).  Figure 9 showed examples of haptic positions at 2 years after surgery. Six percent of the eyes (3/46) had four haptics located in the ciliary sulcus (Figure 9(a)), 70% of the eyes (32/46) had four haptics located in the ciliary processes (Figure 9(b)), and 24% eyes (11/46) had haptics located either in the ciliary processes or in the ciliary sulcus.

## 7. Discussion

The present study reported 2-year results after the ICL V4c implantation in a group of high-myopic patients. Refractive outcomes, anterior chamber changes, and haptic position in the posterior chamber were evaluated, which could help to better understand the latest generation of ICL.

The efficacy and safety indexes of 1.03 and 1.24 in this study are comparable with previous studies; for example, the indexes were 1.00 and 1.01 at 6 months in Alfonso's study [3], 1.03 and 1.13 at 6 months in Shimizu's study [2], and 1.00 and 1.04 at 12 months in Lisa's study [4]. Compared with the above studies, the mean postoperative SE was more myopic in the current study. The following reasons are listed. Firstly, this study population was mostly consisted of high myopic (SE of −8.00 D or higher) adults. The mean preoperative SE was −14.62 D, extremely higher than the abovementioned studies with the mean SE of −7.30 D to −8.80 D [2–4]. Owing to the high percentage of super-high myopia (23% over −18.00 D) in the study group, the residual myopic diopters existed for they were beyond the ICL V4c correction range. Secondly, the mean postoperative astigmatism was also higher in this study, and some of the eyes had astigmatism undercorrected for different reasons as we described in Patients and Methods, which lead to the existence of residual cylindrical diopters in our study. Still and all, the predictability results showed that 98% of the eyes had their attempted SE within ±1.00 D of the intended correction. Therefore, despite the residual refractive diopters, the correction efficiency is satisfying. The extremely high diopters were significantly decreased with no vision threatening complications occurred; 75% had UDVA of 20/25 or higher, and 75% of the eyes gained more lines of CDVA after operation, suggesting efficacy and safety of ICL V4c implantation for correcting super-high myopia.

In another study, we found that ICL implantation is safe and effective for the correction of residual refractive error after corneal refractive surgeries [5]. Conversely, if the cornea condition fits the operation indications, corneal laser surgeries would be suitable to treat the residual diopters after ICL implantation. Sánchez-Galeana et al. reported that LASIK or PRK can be used to treat the residual refractive error following posterior chamber phakic IOL implantation [6]. Zaldivar et al. reported that combined posterior chamber phakic IOL implantation with LASIK was an effective and predictable method for correcting myopia from −18 to −35 D [7]. Comparative studies found no significant difference between the outcomes of the conventional ICL and the hole ICL [8–10]. The combination of ICL V4c with corneal refractive surgery to treat super-high myopia could be further explored.

The most common concerns regarding ICL implantation are cataract formation and secondary glaucoma [11]. None of the eyes in the present study developed cataract, and no IOP increase was observed at 2 years. The results are consisting with previous studies [2–4]. A few studies reported that anterior subcapsular opacification rather than clinically significant cataract might happen after ICL V4c implantation, and the rate (approximately 3%) was half of that in V4b [12, 13]. The insertion of an ICL will reduce the aqueous humor circulation and cause a metabolic disturbance of the crystalline lens [14]. The innovative central hole design of ICL V4c increases aqueous humor flow onto the crystalline lens, potentially decreases the risk of cataract formation after ICL V4c implantation [15]. Sanders estimated through Kaplan–Meier analyses that about 7% of the eyes develop anterior subcapsular opacification at 7 years after ICL implantation, and only 2% will progress to visually significant cataracts [16]. Longer-term complications after ICL V4c implantation need to be further observed.

Vault was defined as the central distance between the posterior surface of the ICL V4c and the anterior surface of the crystalline lens. Excessive low vault increases the risk of cataract formation for the artificial lens might touch the crystalline lens. On the contrast, extremely high vault might induce angle closure glaucoma. In Gonvers's study regarding V3 and V4, they suggested that a vault greater than 150 *μ*m (at least 90 *μ*m) was able to avoid the lens contact and prevented cataract formation [17]. The vault was pretty stable between time points in this study, ranging from 130 *μ*m to 810 *μ*m at 2 years. There is one eye in our study that had the vault (130 *μ*m) lower than 150 *μ*m but higher than 90 *μ*m and currently without developing the anterior subcapsular opacification. Pupil size/movement, crystalline lens thickness changes, and the ICL haptics position were considered to be influential factors of vault [18–20]. We found in this study group that the higher the vault, the narrower the anterior chamber angle width, and the smaller the anterior chamber volume at postoperative 2 years. A recent study also reported a weak relationship between the anterior chamber angle width and vault at 18 months after ICL V4c implantation [21]. It is obvious that the higher the vault, the more the implanted lens pushes forward the iris to the cornea, the more the reduction of the anterior chamber space, and therefore, the more the attention should be paid to the intraocular pressure changes.

Anterior chamber segment changes after ICL implantation were evaluated using different methods, such as Fourier-domain optical coherence tomography (FD-OCT) [22] and UBM [23]. We used Pentacam in the current study and found that the anterior chamber angle width decreased to 62% at 2 years. Eissa et al. also found that angle width in degree decreased to 64% of the preoperative value at 18-month follow-up time point using the Scheimpflug tomography imaging system, from preoperative 40.14 degree to 25.49 degree [21]. Similarly, no correlation was found between anterior chamber angle width and IOP after operation in their study. The narrowing of anterior chamber angle after ICL implantation usually achieves stability in 1 month after ICL implantation. For example, in 48 eyes of 29 patients with high myopia treated with the traditional ICL, Chung et al. reported that trabecular-iris angle decreased by 42% at 1 month, and no significant progressive changes were observed thereafter over a mean follow-up of 33 months [23]. Fernández-Vigo et al. reported that trabecular-iris angle measured with FD-OCT reduced by 34% to 42% at 1 month after ICL V4c implantation and then remained stable at 3 months [22]. The above evidences suggested that the narrowing of the anterior chamber angle after ICL V4c implantation had little impact on intraocular pressure, and the stable status could be achieved in 1 month. A carefully monitoring of anterior chamber angle and IOP is required in the early postoperative period. However, despite that the longer period results suggested a great stability of anterior chamber angle, regular visits are essential as the anterior chamber space decreases with age, especially after 40 years of age [24].

The abnormal position of ICL in the posterior chamber is the main cause of cataract and glaucoma [16, 25]. Ultrasound biomicroscopy (UBM) is a unique way to noninvasively evaluate the position of ICL in the posterior chamber and its relation with adjacent tissues, such as the ciliary body, crystalline lens, and iris. There were very few studies which evaluated the haptics position of ICL V4c in the posterior chamber. A recent study reported that the haptics of V4 was located in the ciliary sulcus in 112 (79%) of their 142 eyes at 6 months, and at least one haptic was found to locate on the lens periphery and zonules in 21% of the eyes [26]. The haptics of ICL V4c were mostly located in the ciliary processes in our study and did not increase the risk of anterior subcapsular opacification or high IOP. And the vault was stable during the 2 years, indicating a stable position of the ICL V4c lens.

In conclusion, implantation of ICL V4c is an effective, safe, and predictive option for correcting high and super-high myopia. Pentacam and UBM can provide detailed anatomical information to monitor the ICL V4c position and its relationship with adjacent tissues. The decreased anterior chamber space after ICL V4c implantation did not induce intraocular pressure increasing during the 2-year follow-up.

## Figures and Tables

**Table 1:** Patient demographic data and ICL V4c characteristics.

Parameters	Mean ± SD	Range (min, max)
Age (year)	30.87 ± 8.03	20.00, 45.00
Spherical equivalent (D)	−14.62 ± 4.29	−25.75, −8.00
Refractive cylinder (D)	−1.82 ± 1.22	−5.75, 0.00
CDVA (decimal)	0.83 ± 0.24	0.20, 1.20
ICL V4c size (mm)	12.93 ± 0.42	12.10, 13.70
ICL V4c power (D)	−14.10 ± 2.85	−18.00, −8.00

ICL V4c = Visian Implantable Collamer Lens with a central hole; SD = standard deviation; D = diopters; CDVA = corrected distance visual acuity; mm = millimeter.

## Data Availability

The data could be found in the Department of Ophthalmology, Eye and ENT Hospital of Fudan University, Myopia Key Laboratory of the Health Ministry, Shanghai.
